# Studies on two polyherbal formulations (ZPTO and ZTO) for comparison of their antidyslipidemic, antihypertensive and endothelial modulating activities

**DOI:** 10.1186/1472-6882-13-371

**Published:** 2013-12-26

**Authors:** Nauman Aziz, Malik Hassan Mehmood, Anwarul-Hassan Gilani

**Affiliations:** 1 Department of Pharmacology, Faculty of Pharmacy, University of Karachi, Karachi, Pakistan; 2 Natural Product Research Division, Department of Biological and Biomedical Sciences, The Aga Khan University Medical College, Karachi 74800, Pakistan; 3 Department of Pharmacy, College of Health Sciences, Mekelle University, Mekelle Ethiopia

**Keywords:** Polyherbal formulation, Endothelial dysfunction, Antihypertensive antidyslipidemic, Antioxidant

## Abstract

**Background:**

Cardiovascular disorders (CVDs) are the leading cause of disease burden worldwide. Apart from available synthetic drugs used in CVDs, there are many herbal formulations including POL-10 (containing 10 herbs), which have been shown to be effective in animal studies but POL-10 was found to cause tachycardia in rodents as its side effect. This study was designed to modify the composition of POL-10 for better efficacy and/or safety profile in CVDs.

**Methods:**

To assess the antidyslipidemic, antihypertensive and endothelial modulatory properties of two herbal formulations, (ZPTO and ZTO) containing Z: *Zingiber officinalis*, P: *Piper nigrum,* T: *Terminalia belerica* and O: *Orchis mascula*, different animal models including, tyloxapol and high fat diet-induced dyslipidemia and spontaneously hypertensive rats (SHR) were used. Effect on endothelial function was studied using isolated tissue bath set up coupled with PowerLab data acquisition system. The antioxidant activity was carried out using DPPH radical-scavenging assay.

**Results:**

Based on preliminary screening of the ingredients of POL-10 in tyloxapol-induced hyperlipidemic rats, ZPTO and ZTO containing four active ingredients namely; Z, P, T and O were identified for further studies and comparison. In tyloxapol-induced hyperlipidemic rats, both ZPTO and ZTO caused significant reduction in serum triglyceride (TG) and total cholesterol (TC). In high fat diet-fed rats, ZPTO decreased TC, low-density lipoproteins cholesterol (LDL-C) and atherogenic index (AI). ZTO also showed similar effects to those of ZPTO with additional merits being more effective in reducing AI, body weight and more importantly raising high-density lipoproteins. In SHR, both formulations markedly reduced systolic blood pressure, AI and TG levels, ZTO being more potent in reversing endothelial dysfunction while was devoid of cardiac stimulatory effect. In addition, ZTO also reduced LDL-C and improved glucose levels in SHR. In DPPH radical-scavenging activity test, ZTO was also more potent than ZPTO.

**Conclusion:**

The modified formulation, ZTO was not only found more effective in correcting cardiovascular abnormalities than ZPTO or POL-10 but also it was free from tachycardiac side-effect, which might be observed because of the presence of *Piper nigrum* in ZPTO.

## Background

Cardiovascular diseases (CVDs) carry a major health burden, affecting humankind [1]. Hypertension, dyslipidemia, endothelial dysfunction and oxidative stress are the major pathologies involved in CVDs and impose a great risk [2]. Apart from the synthetic drugs used for controlling these pathologies, a number of traditional herbal medicines have been in use in various cultures around the world. A polyherbal formulation containing 10 herbs (POL-10), has been used in folk medicine of Pakistan for cardiac ailments. In animal studies, POL-10 has been reported to possess antihypertensive, endothelial modulating and antidyslipidemic activities in SHR [3]. However, it caused an increase in the heart rate and body weight in treated animals, which are considered harmful side-effects in CVDs. This study was designed to modify the composition of POL-10 for better efficacy and/or safety profile for its use in CVDs. Based on preliminary screening of the ingredients of POL-10 in tyloxapol-induced hyperlipidemic rats, two formulations containing four active ingredients namely; Z: *Zingiber officinalis* (Ginger), P: *Piper nigrum* (Black pepper), T: *Terminalia belerica* (Balela) and O: *Orchis mascula* (Saalab misri), labeled as ZPTO and three ingredients (excluding P), labeled as ZTO were identified for further studies to compare their effect on high fat-diet induced dyslipidemia, high blood pressure in spontaneous hypertensive rats (SHR), body weight, endothelial function in isolated vascular preparation of rats, and antioxidant effect using DPPH radical-scavenging assay.

It was also interesting to study the polyherbal formulations as some of the ingredients of these selected formulations, *T. belerica*[4,5]; *Z. officianlis* and *P. nigrum*[6] have been reported to have synergistic activities when used in combination with other medicinal herbs. Based on a cultural belief in south Asia, the compound preparations are considered more effective and safer than the single plant [7], which was supported by recent studies on polyherbal formulations including trifala, in which, *T. belerica* (also part of current study) is the essential ingredient [4].

The available data on the individual components of selected polyherbal formulations; *Z. officinalis*[8,9], *P. nigrum*[10,11]*, T. belerica*[5,12] and *O. mascula*[13] provide an indirect evidence for their effectiveness in cardiovascular abnormalities using non-specific experimental models. In addition, *Z. officinalis*[14,15] and *O. mascula*[16] have also been studies individually in our lab using a set of *in-vitro* assays and animal models for their efficacy in cardiovascular disorders. However, there is hardly any comprehensive study available indicating the effectiveness of the selected combinations of medicinal herbs in CVDs with synergistic and/or side effects neutralizing potential, as the intake of single ingredient alone at high dose for longer duration could be otherwise harmful [17].

## Methods

### Plant material and extraction

All ingredients (*Zingiber officinalis* (Z), *Piper nigrum* (P), *Terminalia belerica* (T), *Orchis mascula* (O) of ZPTO were purchased from a local herbal store in Karachi, Pakistan. The samples were authenticated by Mr. M. Afzal Rizvi, a botanist at the Hamdard University, Karachi. The individual samples were deposited in the herbarium of Natural Products Research Division at the Department of Biological and Biomedical Sciences, The Aga Khan University, Karachi, Pakistan with respective vouchers numbers assigned as ZO-RZ-03-06-75, PN-FT-03-06-72, TB-FT-03-06-74 and OM-RT-03-06-70. The components of ZPTO were ground to fine powder separately and finally mixed together in equal proportion (50 g each). For chronic study, ZPTO and its slightly modified form ZTO, which was prepared by excluding P from ZPTO, in powdered form, were mixed in diet. For free radical scavenging and acute toxicity tests, ZPTO and ZTO were soaked in aqueous-methanol (30:70) for three days with occasional shaking. It was filtered through a muslin cloth and then through a Whatman qualitative grade No.1 filter paper [18]. This procedure was repeated three times and the filtrates were combined and evaporated on rotary evaporator to obtain the thick dark brown crude extract, yielding approximately 10% (wt/wt).

### Drugs and standards

Acetylcholine chloride, cholesterol, cholic acid, phenylephrine, potassium chloride and tyloxapol (triton WR-1339), were obtained from the Sigma Chemical Company, St. Louis, MO, U.S.A. Randox diagnostic kits for serum analysis obtained from Randox Laboratories Ltd., Co. Antrim, UK. All chemicals used were of the highest purity grade. Physiological salt solutions were prepared fresh in distilled water on the day of experiment, while stock solutions of all the drugs and extract were made in distilled water/saline and stored at −20°C and the dilutions were prepared fresh on the day of the experiment.

### Animals

Spontaneously Hypertensive Rats (Strain: SHR/NCrlBR; hypertensive, non-stroke) and its normotensive controls Wistar Kyoto Rats (WKY) were imported from Animal Resource Centre, Australia at the age of 4 weeks. While other animals such as, Sprague–Dawley (SD) rats (170–200 g) and Balb/c mice (20–25 g) were sourced locally and housed at the animal house of the Aga Khan University. Animals were kept in their respective cages with sawdust where needed (renewed after every 48 h), maintained at 23–25°C and had free access to food and water. Experiments performed complied with the rulings of the Institute of Laboratory Animal Resources, Commission on Life Sciences, National Research Council [19] and this study was part of the PhD proposal of Dr. Nauman Aziz, approved by the Board of Advanced Studies and Research, University of Karachi, Karachi, Pakistan.

### Preparation of diets

The diets (A-D) were prepared as;

A. *Normal diet:* The normal diet was prepared as described previously by Harkness [20], at the animal house of the Aga Khan University (AKU), Karachi. The standard diet consisting of flour (5 Kg), chokar (5 Kg), molasses (150 g), salt (75 g), nutrivet L (33 g), potassium meta bisulphate (15 g), oil (500 g), fish meal (2.25 Kg) and powdered milk (2 Kg) for a total of about 13 Kg of the food material.

B. *Treatment diets:* ZPTO or ZTO (3% w/w) mixed in diet A.

C. *High fat/Atherogenic diet:* Cholesterol (2% w/w), cholic acid (0.5% w/w) and butter fat (5% w/w) were added to A [21].

D. *Treatment diet:* ZPTO or ZTO (3% w/w) mixed in diet C.

All measures were taken to ensure the uniform mixing of additives and ZPTO or ZTO powder in dry ingredients of diet before kneading.

### Pharmacological parameters

#### Study on tyloxapol-induced hyperlipidemia

An earlier described animal model of tyloxapol-induced hyperlipidemia [22] was followed with slight modifications. Male Sprague–Dawley (SD) rats weighing 160–180 g were randomly divided into 14 groups (6 in each), group 1 and 2 were given diet A (normal diet) and group 3 and 4 were given diet B (normal diet with ZPTO or ZTO), while animals in groups 5–8 were given normal diet with Z, P, T or O. The remaining six groups were administered remaining 6 components namely; *Aristolochia rotunda, Cinnamomum officinalis*, *Emblica officinalis*, *Matricaria chamomile*, *Piper longum* and *Plumbago zeylanica*, of original polyherbal formulation POL-10. After 10 days of the treatment, all animals were fasted for 7 h and group 1 received saline (10 ml/kg; i.p.), while group 2–14 received tyloxapol (500 mg/kg; i.p.). On the next day, the animals were anaesthetized by diethyl ether and the blood was collected for analysis of serum total cholesterol (TC) and triglycerides (TG) as described in the section of “*Estimation of lipid profile and glucose”*.

### Study on lipid profile, diet consumption and body weight in high fat diet- fed rats

Both ZPTO and ZTO were studied for their effect on high fat diet-induced hypercholesterolemia using a method described by Berroughui et al. [23] with slight modifications after pilot studies. The adult SD rats (12–14 weeks old, weighing 140–160 g) were randomly divided into 4 groups (7–10 animals in each group), group 1 was given diet A (served as normal control) and diet C (atherogenic control) was administered to group 2, while group 3 and 4 were given diet D (treated) for 8 weeks. All animals had free access to water and diet. The diet consumption was monitored daily and the change in body weight was measured weekly for 8 weeks [23]. At the end of 8th week of treatment, animals were fasted 16 h prior to blood collection and samples were analyzed for serum lipids and glucose as detailed in section “*Estimation of lipid profile and glucose”*

### Study on blood pressure, heart rate and lipid profile in SHR

Male Spontaneously Hypertensive Rats (SHR) aged 20–24 weeks, were randomly divided into 3 groups (n =7-8 in each). Group 1 was given diet A (normal diet) and group 2 and 3 were given diet B (normal diet with ZPTO or ZTO) for 8 weeks. Wistar Kyoto Rats (WKY) served as normotensive control and given diet A for same period. The systolic blood pressure was estimated before start and during 8 weeks of the treatment by using a tail-cuff plethysmograph (Model 92, IITC Inc., Woodland Hills, USA), coupled to PowerLab 4/25 data acquisition system coupled to a computer running Chart 5.3 software (ADInstruments, Sydney, Australia). Heart rate was calculated online using cyclic measurements option of the Chart software. Rats were trained daily for ten days before starting the experiment, in terms of the measurement of blood pressure. After acclimatization with the blood pressure measurement procedure, 6–8 readings of systolic blood pressure of each conscious animal were recorded and median values calculated after discarding maximum and minimum. The systolic blood pressure was measured at 0, 4 and 8 weeks of the treatment. The described non-invasive blood pressure measurement is reported to have 96% correlation with direct blood pressure [24]. All animals had free access to water and diet throughout study duration. At the end of 8th week of treatment, the animals were fasted 16 h prior to blood collection and samples were analyzed for serum lipids and glucose as mentioned in section “*Estimation of lipid profile and glucose”*

### Study on vascular reactivity in rat aortae of SHR

At the end of the treatment, SHR and WKY were fasted for 16 h, anaesthetized with diethyl ether by inhalation, blood was collected via cardiac puncture and serum was analyzed for lipid profile and glucose. Thoracic aortae were isolated and studied for endothelial reactivity. The descending thoracic aortae were transferred immediately to Kreb's buffer (composition in mmol/L: NaCl 118.4, KCl 4.7, CaCl_2_ 2.5, KH_2_PO_4_ 1.2, MgSO_4_ 1.2, NaHCO_3_ 25, and glucose 11), cleaned of periadventitial tissue, and were cut transversally into ring segments (3 mm in length). Each ring was placed in a tissue bath filled with Kreb's buffer (37°C) bubbled with carbogen (95% O_2_ and 5% CO_2_), and attached to a force transducer (model FORT100) coupled to a Trans-bridge (model TBM4M, World Precision Instruments, Hertfordshire, UK) and PowerLab data acquisition system for measurement of the isometric tension. Aortic rings were allowed to equilibrate at least for 60 min at a resting tension of 2 g with changes of buffer every 15 min. When the isometric tension had stabilized, inhibitory concentration-response curves (CRCs) of acetylcholine (ACh) 1 × 10^-9^ -10^-5^ mol/L were constructed against contractions induced with submaximal dose of phenylephrine (1 × 10^-6^ mol/L; PE) [25].

### Estimation of lipid profile and glucose

The blood was collected in vacuutainer by cardiac puncture from anaesthetized rats fasted overnight. The serum was separated after centrifugation at 3000 rpm for 10 min. The serum lipids and glucose were assayed enzymatically using commercially available kits of Randox Laboratories, Serum total cholesterol (TC), high-density lipoproteins (HDL-C), triglyceride (TG) and glucose were determined by methods described by manufacture (Randox Laboratories Ltd., Co. Antrim, UK). Low-density lipoproteins cholesterol (LDL-C) was estimated indirectly by using formula: LDL = Total Cholesterol – HDL – TG/5). Atherogenic index was calculated using formula: Atherogenic index = TC-HDL/HDL) [26].

### Free radical scavenging activity

With slight modifications in an earlier method of Misbah et al. [27], a 0.1 mM solution of 1,1-diphenyl-2-picryl-hydrazil (DPPH) radical in methanol was prepared and 1 ml of this solution was added to 3 ml of the extract solution in methanol at different concentrations and then absorbance was measured after 30 min at 517 nm. Decrease in the absorbance of DPPH solution indicates an increase in DPPH radical-scavenging activity. The percent DPPH radical-scavenging activity was calculated by equation:

$$\begin{array}{l} \%\;\mathrm{DPPH}\;\mathrm{radical}\;\mathrm{scavenging}\\ \;=\left(1-\frac{\mathrm{Control}\;\mathrm{Absarbance}-\mathrm{Sample}\;\mathrm{Absorbance}}{\mathrm{Control}\;\mathrm{Absorbance}}\right)\\ \;\times 100 \end{array}$$

The DPPH solution was taken as the control. The CRCs were plotted as the concentrations of extracts in μg/ml against percent of free radical scavenging activity for the calculation of median effective concentrations (EC_50_) values along with 95% confidence interval.

### Acute toxicity test

BALB/c mice were divided into 4 groups of 5 in each and given increasing doses of the crude extracts of ZPTO and ZTO (1, 2 and 3 g/kg) in a volume of 10 ml/kg. A negative control group of mice was administered saline (10 ml/kg, p.o.). Animals had free access to food and water and kept under observation for 24 h for mortality assessment [28].

### Statistical analysis

All the data expressed as mean ± SEM and the EC_50_ values are presented as geometric mean with 95% confidence intervals (CI). For comparison between means of two groups unpaired Student’s *t*-test was used, while One-way Analysis of Variance (One-way ANOVA) was used to compare the differences in means of more than two groups with control, followed by Dunnett’s multiple comparison test. The concentration-response curves (CRCs) were analyzed by using non-linear regression analysis. Two-way ANOVA followed by Bonferroni’s post-test correction was used for multiple comparisons of CRCs with the respective control. P-values less than 0.05 (p < 0.05) were considered as statistically significant. All the graphs, calculation and statistical analyses were performed using GraphPad Prism software version 4.00 for Windows, (GraphPad Software, San Diego California USA, http://www.graphpad.com).

## Results

### Effects of ZPTO, ZTO and their components on tyloxapol-induced hyperlipidemia

In an attempt to compose a new formulation with better efficacy and/or safety profile, individual ingredients of POL-10, a previously studied polyherbal formulation [3], were screened in this model. Administration of tyloxapol to rats caused an increase in serum triglycerides (TG) to 6295 ± 499.30 vs. 71.56 ± 2.69 mg/dl (p < 0.001; n = 8-10) and total cholesterol (TC) to 531.6 ± 30.01 vs. 89.51 ± 2.31 mg/dl (p < 0.001; n = 8-10) as compared to untreated animals. Out of 10 ingredients of POL-10, only 4 showed positive effect on serum TG and TC levels, while remaining 6 components were found inactive in this model (data not shown). Pretreatment of animals with individual plants of the newly designed formulation ZPTO caused a significant (p < 0.01) reduction in TG with respective values of 3075 ± 119 mg/dL (*Z. officinalis*), 3536 ± 897.2 mg/dL (*P. nigrum*), 3588 ± 150.7 mg/dL (*T. belerica*) and 2823 ± 114 mg/dL (*O. mascula*) compared to 6295 ± 499.30 mg/dL (tyloxapol-administered control). Similarly, both formulations ZPTO (2336 ± 574 mg/dL) and ZTO (2499 ± 633.6 mg/dL) also produced a marked (p < 0.01) reduction on serum TG levels compared to tyloxapol administered control. Both ZPTO and ZTO were found more effective (at the 1/4^th^ and 1/3^rd^ doses) in reducing TG when compared to the individual ingredients.

In tyloxapol-induced hypercholesterolemia, pretreatment of the animals with *Z. officinalis* (295.4 ± 99.92 mg/dL), *O. mascula* (277.6 ± 84.74 mg/dL), *P. nigrum* (373 ± 66.09 mg/dL), ZPTO (319.3 ± 61.29 mg/dL) and ZTO (341.9 ± 70.91 mg/dL) significantly (p < 0.01) prevented tyloxapol-induced increase in TC levels. However, *T. belerica* (466.6 ± 23.69 mg/dL) was found devoid of any effect (p > 0.05) on TC levels. Both ZPTO and ZTO showed similar degree of reduction in TC levels compared to their individual components but at 1/4^th^ and 1/3^rd^ of the doses.

### Effects of ZPTO and ZTO on lipid profile, diet consumption and body weight in high fat diet- fed rats

An atherogenic diet fed for 8 weeks, caused a significant increase in TC, LDL-C, atherogenic index and glucose levels along with an increase in body weight of SD rats compared to the data obtained from animals on normal diet. Supplementation of 3% ZPTO and 3% ZTO to atherogenic diet fed rats, caused a significant (p < 0.001) reduction in TC, LDL-C, atherogenic index and daily diet consumption. However, treatment with ZTO offered additional benefits in terms of raising HDL-C. ZTO showed comparatively better (p < 0.05) effect on atherogenic index than ZPTO, while ZPTO caused stronger effect (p < 0.5) on glucose levels.

When studied for their effect on weight gain in obese rats fed on atherogenic diet, ZPTO had no effect, however, a marked reduction (p < 0.01) was observed in the body weight of animals treated with ZTO. Both, ZPTO and ZTO did not show any effect on TG levels (Table 1).

**Table 1:** Effects of polyherbal formulations (ZPTO and ZTO) on serum lipids and glucose levels in atherogenic diet-fed Sprague Dawley rats

**Parameters**	**ND**	**AD**	**AD + 3% ZPTO**	**AD + 3% ZTO**
Total Cholesterol (mg/dl)	74.62 ± 3.9	390.5 ± 3.09 **###**	110.3 ± 12.14***	110.7 ± 10.24***
HDL-C (mg/dl)	38.54 ± 1.7	27.82 ± 2.97	30.91 ± 3.1	82.6 ± 11.45***
LDL-C (mg/dl)	24.9 ± 2.8	304 ± 35.7 **###**	74.5 ± 9.4***	57.24 ± 10.8***
Triglycerides (mg/dl)	59.7 ± 4.2	65.2 ± 6.4	71.6 ± 11.6	49.5 ± 11.8
Atherogenic Index	0.92 ± 0.12	16.96 ± 3.15**###**	3.53 ± 0.813**	0.7 ± 0.155***
Glucose (mg/dl)	95.6 ± 7.2	147.5 ± 4.8 **###**	94.18 ± 6.96***	130.3 ± 3.15**
Daily diet consumption (g/Kg/day)	186.5 ± 11.8	161.8 ± 12.4**#**	78.9 ± 10.8***	121.9 ± 7.1***
% Change in body weight	33.33 ± 1.6	56.8 ± 1.97**###**	53.2 ± 0.8	46.51 ± 1.61**

Normal Diet (ND) administered Sprague Dawley rats (SD), Atherogenic Diet (AD) administered SD rats; Polyherbal formulations, ZTO and ZPTO are composed of *Zingiber officinalis* (Z), *Terminalia belerica* (T), *Orchis mascula*, or/and *Piper nigrum* (P)*;* Values shown are Mean ± S.E.M. of 7–10 determinations.

^**#**^p <0.05 and ^**###**^p <0.001 compared to ND (Student *t*-test).

**p <0.01 and ***p <0.001 compared to AD (One-way ANOVA followed by Dunnett’s multiple comparison-test).

### Effects of ZPTO and ZTO on blood pressure and lipid profile in SHR

Treatment of SHR with 3% ZPTO or 3% ZTO for 8 weeks significantly reduced systolic blood pressure as compared to untreated hypertensive control. Treatment with ZPTO and ZTO significantly (p < 0.01) lowered systolic blood pressure in SHR. Treatment of animals with ZPTO also increased (p < 0.001) heart rate in SHR, while ZTO did not (p > 0.05) alter it. No change in HDL-C levels was noticed in serum samples of animals treated with ZPTO or ZTO, while a significant (p < 0.05) reduction was observed in LDL-C levels of animals treated only with ZTO. Both preparations produced a significant reduction in TG levels (p < 0.001) and atherogenic indices (p < 0.01) when compared with SHR control. On glucose levels, treatment of ZPTO did not (p > 0.05) produce any effect, while ZTO administration significantly (p < 0.01) improved glucose levels in SHR when compared with untreated SHR as shown in Table 2.

**Table 2:** Effects of polyherbal formulations (ZPTO and ZTO), on blood pressure, serum lipids and glucose levels in SHR

**Parameters**	**WKY**	**SHR**	**SHR + 3% ZPTO**	**SHR + 3% ZTO**
Systolic BP (mmHg)	141.7 ± 2.4	200.9 ± 10.4**###**	174.4 ± 4.49**	173.3 ± 5.4**
Heart Rate (BPM)	312.0 ± 22.2	342.9 ± 6.1**#**	383 ± 18.9***	349.1 ± 23.9
Total Cholesterol (mg/dl)	117.3 ± 5.1	93.3 ± 8.1**#**	76.8 ± 11.2	82.83 ± 5.3
HDL-C (mg/dl)	98.28 ± 5.8	70.15 ± 7.7**##**	66.5 ± 8.3	71.14 ± 4.5
LDL-C (mg/dl)	19.1 ± 3.4	23.13 ± 3.8	18.4 ± 3.6	12.3 ± 3*
Triglycerides (mg/dl)	82.85 ± 3.7	98.0 ± 4.4**#**	60.5 ± 5.3***	64.2 ± 6.7***
Atherogenic Index	0.20 ± 0.04	0.37 ± 0.09**###**	0.15 ± 0.04**	0.17 ± 0.03**
Glucose (mg/dl)	113.4 ± 6.1	88 ± 13.6**#**	80.8 ± 7.8	130.3 ± 17.6**

Wistar Kyoto Rats (WKY), Spontaneously Hypertensive Rats (SHR).

Polyherbal formulations, ZTO and ZPTO are composed of *Zingiber officinalis* (Z), *Terminalia belerica* (T), *Orchis mascula* or/and *Piper nigrum* (P).

Values shown are Mean ± S.E.M. of 7–8 determinations.

^**#**^p <0.05; ^**##**^p <0.01; ^**###**^p <0.001 compared to WKY (Student t-test).

*p <0.05; **p <0.01; ***p <0.001 compared to SHR (One-way ANOVA followed by Dunnett’s multiple comparison-test).

### Effect of ZPTO and ZTO on vascular reactivity in isolated rat aortae of SHR

**Figure 1:**
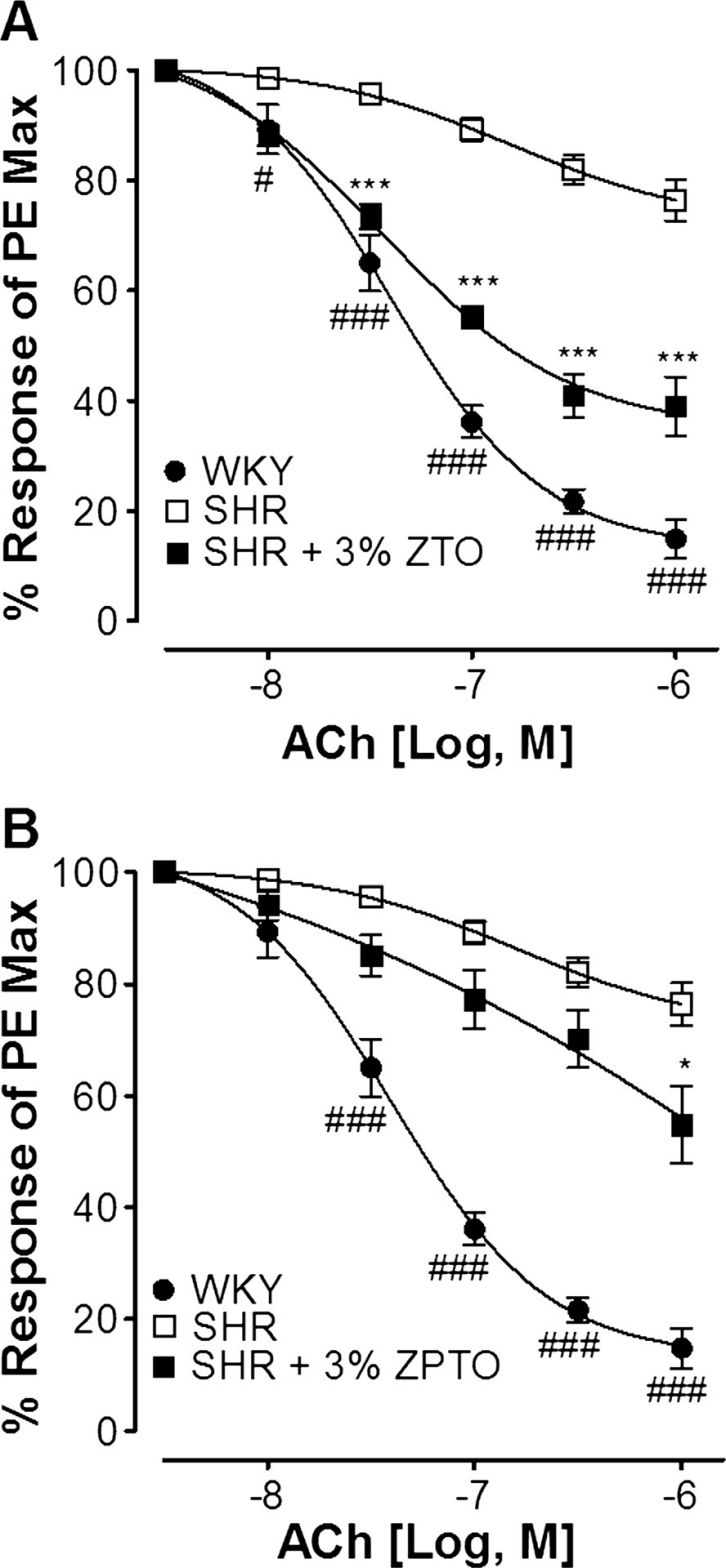
**Dose–response curves showing the vasorelaxant effects of acetylcholine on phenylephrine-induced (PE: 1 μM) vasoconstriction in aortae isolated from Spontaneously hypertensive rats (SHR) and Wistar Kyoto rats (WKY) and SHR treated with 3% ZTO (A) and ZPTO (B) for the period of two months.** Curves represent mean ± S.E.M of responses from 5–10 animals. (#p <0.05, ###p <0.001 showing comparison of WKY with SHR, student *t-test* *p <0.05, ***p <0.001 showing comparison of SHR + 3% ZTO or ZPTO with SHR; Two-way ANOVA, Bonferroni post-test).

Treatment of animals with 3% ZPTO or 3% ZTO for 8 weeks improved the acetylcholine (ACh)-mediated endothelial-dependent relaxation of PE-induced contractions in isolated aortae compared with those of untreated controls. The comparative CRCs of ACh-mediated endothelial-dependent relaxation in the aortae of different groups of animals are presented in Figure 1. The aortic tissues of animals treated with ZTO produced greater relaxant response to ACh application with an EC_50_ value of 0.06 μM (0.03-0.07, n = 7-10) as compared to ZPTO [0.25 μM (0.10-0.29, n = 7-10)], indicating a stronger effect of ZTO on vascular reactivity (Figure 1).

### Antioxidant activity

The crude methanolic extracts of ZPTO and ZTO were studied for their antioxidant activity in DPPH free radical scavenging assay. Both formulations showed a significant effect with EC_50_ values (95% CI; n = 3-6) and R^2^ (a square of correlation coefficient, its value close to 1 shows better correlation between concentration and activity and is also expressed as goodness of concentration curve fitting) as ZPTO: 11.44 μg/mL (6.53-20.04; n = 3-6, 0.991) and ZTO: 4.33 μg/mL (3.98-4.71; n = 3-6, 0.996). These effects were found similar to that of butyl hydroxytoluene (BHT), used as a positive control, which exhibited antioxidant effect with an EC_50_ value of 2.11 μg/mL (1.97-2.26; n = 3-6, 0.999). It is evident that ZTO is almost three times more potent than ZPTO.

### Acute toxicity test

In acute toxicity studies on mice, ZPTO and ZTO up to 3 g/kg per oral (p.o.) did not produce mortality in 48 h. During chronic treatment for 6–8 weeks, ZPTO caused increased in heart rate of SHR, while ZTO was devoid of this effect. Both ZPTO and ZTO did not cause any death or behavioral changes in normotensive rats and SHR.

## Discussion

A polyherbal formulation (POL-10) reported to exhibit a side-effect, increasing heart rate and body weight in treated animals [3]. The purpose of this study was to modify the composition with better efficacy and/or safety profile for its use in CVDs. Hyperlipidemia is the major risk factor of CVDs. Tyloxapol-induced hyperlipidemia model is widely used for screening and exploring mechanism of action of lipid lowering drugs. In this assay, out of 10 ingredients of POL-10, only four *Zingiber officinalis*, *Piper nigrum*, *Terminalia belerica* and *Orchis mascula* were found active. Therefore, a combination of all four ingredients (ZPTO) was studied in further detail. Furthermore, *P. nigrum* also showed some adverse effects like increase in atherognic index and triglyceride levels in SHR. A second combination, ZTO (without *P. nigrum*) was included in the study as the improved version.

In normotensive SD rats, administration of tyloxapol to rats at the dose of 500 mg/kg is known to cause drastic increase in serum TG and TC levels due to enhanced hepatic cholesterol synthesis particularly by increase in HMG Co-A activity [29] and by the inhibition of lipoprotein lipase responsible for hydrolysis of plasma lipids [30]. In this model of hyperlipidemia, both ZPTO and ZTO showed a significant reduction in TG and TC levels with better efficacy than the parent formulation, POL-10 [3]. Similarly, they were more effective (1/4th to 1/3rd of the dose) in reducing TG levels when compared with its individual ingredients, indicating synergistic effect of these formulations on TG levels. These results also showed that the new formulations possess lipid-lowering effects possibly involving inhibition of lipid biosynthesis.

High fat/atherogenic diet containing high cholesterol and cholic acid induces endothelial dysfunction, atherosclerosis [31] and increases oxidative stress by increasing the expression of oxidation-sensitive genes such as Elk-1 and p-CREB [32]. It is also known that high cholesterol diet containing cholic acid increases TC, LDL-C, atherogenic index and decreases HDL-C by enhancing intestinal absorption and secretion and decreasing catabolism of cholesterol [33]. Treatment of rats with ZTO or ZPTO decreased serum TC and LDL-C levels, while no change was observed on TG levels. ZTO was found superior to ZPTO in terms of raising HDL-C. Similarly, ZTO showed better efficacy in reducing atherogenic index, while ZPTO was more effective only in reducing glucose levels, which needs further studies. Considering weight gain and increase in diet consumption leading to obesity, a characteristic feature of high fat diets intake [34], both formulations were also compared for their effect on diet consumption and weight gain. ZPTO was found more effective (P < 0.01) in reducing diet consumption, while only ZTO reduced the weight gain of obese rats on atherogenic diet, showing its additional weight reducing potential, which was not seen in case of ZPTO. These data clearly indicate that in atherogenic diet-fed animal model, ZTO is more beneficial than ZPTO or POL-10 [3]. ZTO also caused reduction in atherogenic index, and raised HDL-C, which are considered better indicators of coronary heart disease risk than individual lipoprotein concentration [35], thus showing additional merit, particularly when lipid lowering chemical drugs have limited success in correcting HDL-C abnormality [36]. SHR model is the most widely used animal model to equate with human essential hypertension, due to its similarities in genetic predisposition to high blood pressure without specific etiology, increased total peripheral resistance without volume expansion, impairment of endothelial function and similar responses to the drug treatments [37]. Vascular endothelium plays an important role in regulation of blood pressure through the synthesis and secretion of a variety of vasoactive substances. In SHR, vascular endothelial dysfunction is caused by a variety of factors, such as hypertension [38], hypertriglyceridemia [39] and excess production of oxidants and/or deficiency of antioxidant systems [40]. Treatment of SHR with ZPTO or ZTO reduced systolic blood pressure, TG and atherogenic index with comparable efficacy, whereas, ZTO was more effective than ZPTO in improving endothelial dysfunction. A major limitation of ZPTO was its ability to increase heart rate in SHR, while ZTO was devoid of this harmful effect. Unlike ZPTO, it also reduced LDL-C and improved hyperglycemia, indicating additional merit of ZTO over ZPTO or POL-10 [3].

High fat diet is known to cause oxidative stress (enzymatic and non-enzymatic) in rats, indicated by elevated levels of thiobarbituric acid reactive substances, conjugated dienes and significantly lowered activities of superoxide dismutase, catalase, glutathione peroxidase, glutathione-S-transferase and reduced glutathione in the liver, heart, kidney, intestine and aorta. Antioxidants effectively prevent such kind of cellular damages [41]. Interestingly, when tested in DPPH assay, ZPTO and ZTO exhibited strong antioxidant activity, which may offer additional benefit in combating the oxidative stress caused by high cholesterol. In SHR, oxidative stress causes disturbance in calcium homeostasis, which is essential for the synthesis and release of nitric oxide (NO) from endothelial cells [42]. Antioxidant properties of new formulations may have played a role in improving NO release and synthesis as indirectly observed in their endothelial modulating potentials. ZTO was found more effective than ZPTO in its antioxidant effect, suggesting another merit of ZTO over ZPTO.

In earlier study, POL-10 was reported to increase heart rate in SHR [3], as was observed with ZPTO but not with ZTO in this study. *Piper nigrum* is the only component, which is making ZTO different from ZPTO and was also a constituent of POL-10. *P. nigrum* and its main alkaloid constituent, piperine have been reported to possess monoamine oxidase inhibitory (MAOI) activity [43]. MAOIs are known to cause inhibition of oxidative deamination of monoamines such as, serotonin, dopamine and norepinephrine and eventually increase their half-lives and activities. Increased levels of norepinephrine, might be responsible for increase in heart rate caused by ZPTO in SHR, though other mechanism(s) cannot be ruled out.

Taking together, the important differences in atherogenic diet-fed rat model were; ZTO was more effective in correcting atherogenic index and HDL-C than ZPTO. Similarly in SHR model, ZTO and ZPTO were found equally effective and relatively more efficacious (p < 0.05) in their antihypertensive action than POL-10 [3], while ZTO was more effective for its positive impact on endothelial vascular reactivity and was also free from tachycardiac side effect, when compared with ZPTO or POL-10 [3]. Moreover, ZTO reduced LDL-C and glucose levels, but not shared by ZPTO. The cumulative better effective profile of ZTO in CVDs might be because of the individual beneficial effects of its components; *Z. officinalis*[14,15,44], *T. belerica*[45,46] and *O. mascula*[16].

The use of polyherbal formulations compared to individual herbs are not only encouraged culturally in the Indo-Pak sub-continent [17], but also supported by evidence possessing the synergistic interactions, thus offering intake of the individual ingredients at lower doses for longer duration, which could otherwise be harmful. Moreover, the selected ingredients of ZPTO and ZTO are known for their synergistic therapeutic action when used with other medicinal herbs in combinations such as, *Trifla* containing *T. belerica* as one of its component, which is a popular herbal formulation for its effectiveness in hypercholesterolemia [4] compared to the individual hypolipidemic potential of *T. belerica*[5]. Similarly, *Trikatu*, a polyherbal formulation corrects deranged lipid in a better way [6] compared to the individual effectiveness of *Z. officinalis*[8,9] and *P. nigrum*[10,11]. The present study also proves better efficacy and safety of *O. mascula* in the form of mixture compared to its individual actions [16].

## Conclusion

These findings suggest that the modified formulation containing: *Zingiber officinalis*, *Terminalia belerica* and *Orchis mascula* was relatively more effective as antidyslipidemic, antihypertensive, antioxidant and endothelial modulating agent, as well as offers better safety profile compared to its parent formulations ZPTO or POL-10. However, further detailed studies on phytochemical, toxicological and pharmacological aspects are required on ZTO, before making it available for clinical trials.

## Abbreviations

ZPTO: (Z) *Zingiber officinalis*; P: *Piper nigrum*; T: *Terminalia belerica*; O: *Orchis mascula*; SHR: Spontaneously hypertensive rats; WKY: Wistar Kyoto rats; SD: Sprague–Dawley Rats; EC50: Effective concentration producing 50% effect; PE: Phenylepherine; ACh: Acetylcholine; CRCs: Concentration-response curves; DPPH: 1,1-diphenyl-2-picryl-hydrazil; AI: Atherogenic index.

## Competing interests

The authors declare that they have no competing interests.

## Authors’ contributions

AHG designed the project, supervised the study and reviewed the final version of manuscript. NA carried out literature search and conducted experimental work. NA and MHM collected and analyzed the data, carried out statistical analysis and drafted the final manuscript. All authors read and approved the final manuscript for publication.

## Authors’ information

NA was a PhD Scholar, Department of Pharmacology, Faculty of Pharmacy, University of Karachi, Karachi, Pakistan; *Currently*, NA is a Country Manager of ADInstruments Australia for Pakistan based in Karachi, Pakistan.

MHM is an Assistant Professor at Natural Product Research Division, Department of Biological and Biomedical Sciences, The Aga Khan University Medical College, Karachi-74800, Pakistan.

AHG is a Professor at Natural Product Research Division, Department of Biological and Biomedical Sciences, The Aga Khan University Medical College, Karachi-74800, Pakistan (on sabbatical leaves); *Currently*, AHG is the Tandem Dean at the College of Health Sciences, Mekelle University, Mekelle, Ethiopia.

## Pre-publication history

The pre-publication history for this paper can be accessed here:

http://www.biomedcentral.com/1472-6882/13/371/prepub
